# The integration of haptic training into the QMUL dental curriculum

**DOI:** 10.1111/eje.12963

**Published:** 2023-10-24

**Authors:** Amitha Ranauta, Ben Audsley, Paul Coulthard

**Affiliations:** ^1^ Institute of Dentistry Queen Mary University of London London UK

**Keywords:** dental curriculum, haptics, simulation training

## Abstract

The Pandemic has challenged clinical dentistry globally with the dental education sector seeking alternative training environments. Virtual reality (VR) is gaining recognition as a valuable tool for training dental students and its use by dental schools around the world is growing. The continuous improvement of haptic VR dental trainers provides a platform where irreversible procedures can be safely and unlimitedly practised. This driver has led to the exploration and investment into virtual technology to improve education outcomes in dental students. The aim of this study was to share the early experience of a dental school in the United Kingdom that has initiated the process of embedding haptics into their simulation training within the dental curriculum. This paper explores the process of embedding and operationalising haptic training within the undergraduate curriculum. Using current knowledge of education pedagogy, the school aligned to an evidence‐based, best‐practice framework which utilised the concept of deliberate practice in the development of the Haptics curriculum which was adaptable and iterative in design. This paper contextualises the implementation of haptic training in a UK dental education setting by providing an outline of the framework used to develop the curriculum. Virtual reality haptics trainers have created unique opportunities and challenges for dental schools. Dental educators have sought to utilise this technology in a structured framework to enhance training.

## 1 INTRODUCTION

The Pandemic has challenged clinical dentistry globally with the dental education sector seeking methods for training which address the management of aerosol‐generating procedures (AGPs) to ensure fit‐for‐purpose dental graduates and postgraduate trainees. The diminution of patient facing training led to training establishments exploring credible alternatives which are safe and sustainable.

In this commentary, the authors present the experience of integration and the impact of Simodont®, (Nissin Inc., Nieuw‐Vennep, Netherlands), haptic virtual simulators into the dental curriculum. The institute of dentistry having been awarded funding from a Charity in 2022 for digital transformation made the decision to invest in digital dentistry. This included the purchase and integration of 42 virtual reality haptic stations and multiple intra‐oral scanners.^1^

Simulation training has always been an integral part of clinical competency‐based dental curriculum. Phantom head and artificial teeth are used in traditional dental simulation training but have limitations which include aerosol generation, cost of disposable items, the necessity of handpiece maintenance and the requirement for direct supervision to deliver feedback.^2^ The aim of this paper is to share the early experience of a UK dental school's experience of integration of haptics into the simulation training curriculum.

## 2 METHODS

The dental school in keeping with healthcare simulation‐based curriculum design set out to include the following components to comply with internationally recognised standards of best practice.^3^, ^4^

**FIGURE 1:**
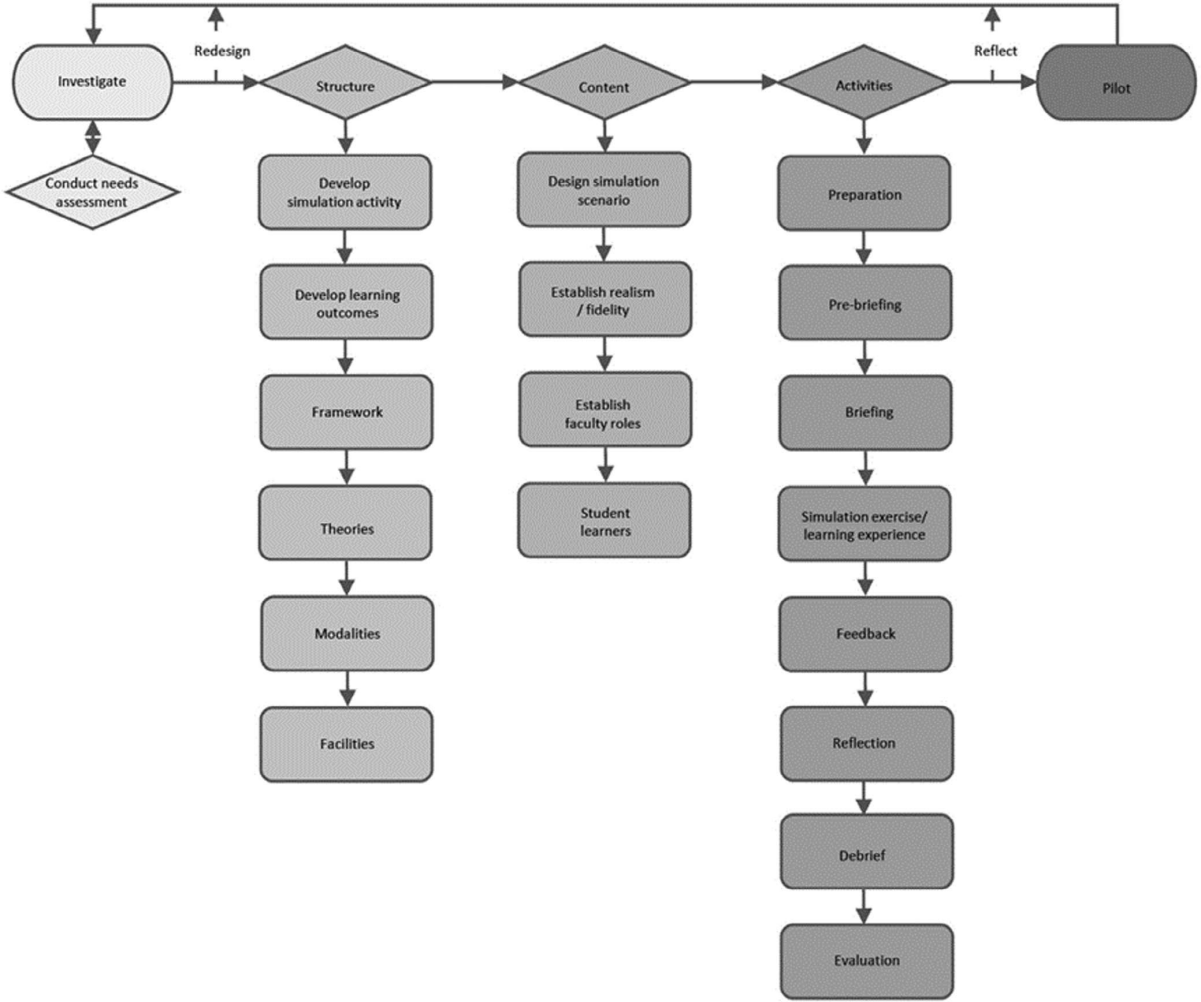
Framework for simulation‐based dental education.^4^

Researchers in Australia have proposed a Framework for Simulation‐based Dental Education (Figure 1).^4^ The Framework development was influenced by instructional design theory for psychomotor skill acquisition, whilst emphasising the value of the collective impact for faculty, curriculum and facilities. The objective of this framework is to guide and support contemporary dental simulation, whilst meeting Faculty requirements for educational quality improvement and to provide students experiences that translate to better education outcomes.

**FIGURE 2:**
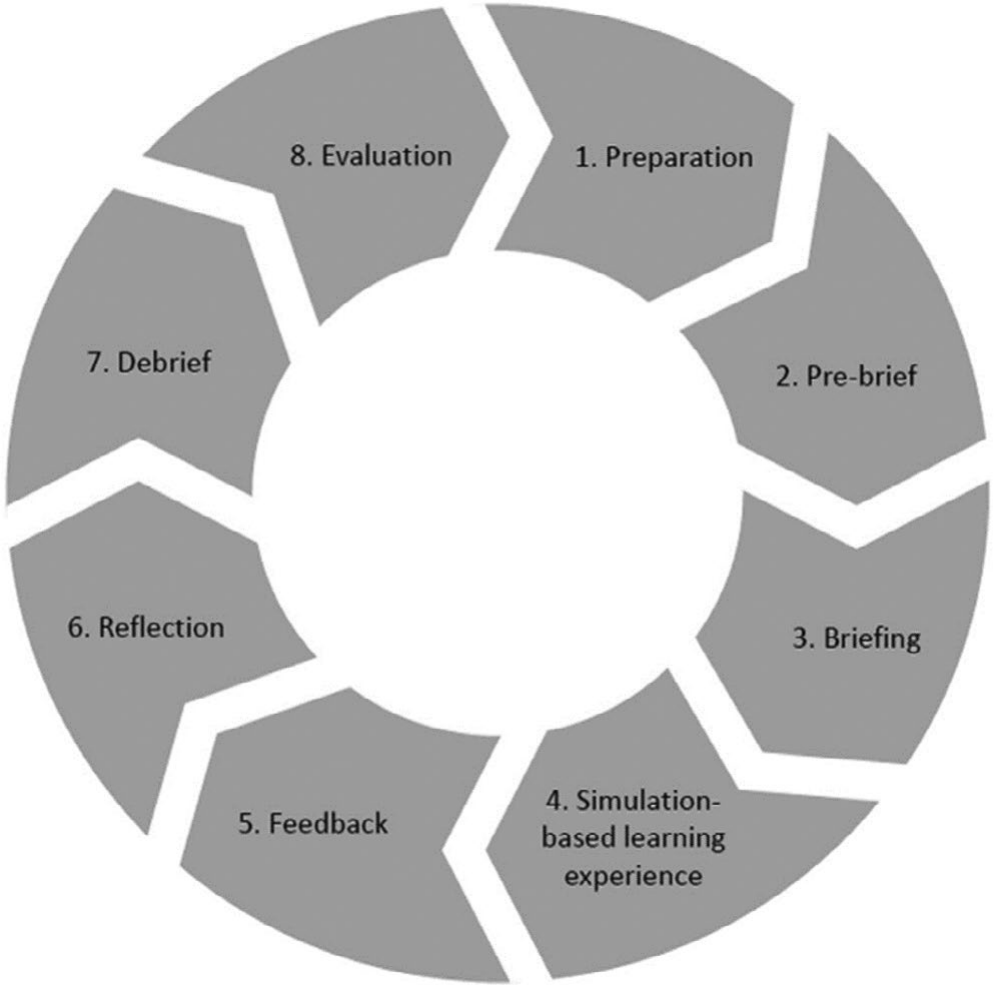
The simulation‐based activity cycle—diagram inspired by Seropian et al.^5^

The application of this framework has given a structure to the development of a haptics curriculum. The launch of the curriculum has used a phased approach which is in keeping with the Simulation based activity cycle (Figure 2).^5^. The existing simulation curriculum utilises the notion of deliberate practice.^6^, ^7^ This is a concept which suggests that expert performance can be drawn to active engagement in deliberate practice, where training (designed and arranged by faculty teachers) is focused on improving particular tasks; which is followed by the provision of immediate feedback, time for problem‐solving and evaluation, and opportunities for repeated performance to refine behaviour.^7^ However, the opportunity to practise in the simulation has been limited due to complex timetables and increasing need for practice in the post pandemic era.

The undergraduate dental curriculum has a hybrid approach which is learning outcomes oriented and has a composition of both competence and integrated learning in the structure. Students typically have responsibility for patient care from year 3 onwards in the 5 years Bachelor of Dental Surgery (BDS) degree in the UK. The 3 years BSc in oral health (Hygiene/Therapy) programme is also integrated with the BDS programme. The key transition point is in year 2 of the BDS programme (and year 1 of BSc), when the majority of the initial simulation training is delivered. It is during this phase that cognitive and technical competence is achieved in preparation for transition into the patient facing care.

In the planning, a decision was made to introduce haptics into this pre‐clinical transition phase. This decision was significant, as this is also the point at which simulation training in Phantom heads with typodont or extracted teeth is established.

This was planned in the preparation phase with a timeframe of approximately 4 weeks which involved regular meetings with the teaching curriculum faculty and the e‐Learning team who had responsibility for installation of hardware and training on the software.

## 3 RESULTS

### 3.1 Preparation and Identifying need

With increasing demands on post‐pandemic clinical training due to increased learner numbers and learners lagging in their training trajectory, the cost factor and changing trends of teaching and assessment, there was a need for the dental school to explore technology‐based teaching and learning software to enhance students' learning.

### 3.2 Education theory

In considering education pedagogy, the school was aligned to an evidence‐based, best‐practice framework.^4^ Deliberate practice described in the literature as a theory of strategic instructional design for purposeful action to acquire psychomotor skills.^8^ This requires explicit learning outcomes, learner focus, consciously assigned degree of task difficulty, structured purposeful repetitiveness, immediate feedback, in‐action reflection, evaluation to work towards mastery learning and increasing degree of difficulty.^9^, ^10^

This was an existing approach utilised in the delivery of the traditional simulation training and was extended to haptics simulation training. The General Dental Council (GDC) regulatory body for dentistry in the UK sets the learning outcomes for the curriculum.^11^ The school was also looking to enhance feedback often recognised by learners in National Student Survey (NSS).^12^

It is envisaged that the haptic 3D VR dental simulator Simodont® will provide objective mechanical force feedback to the learner allowing improvement of manual dexterity skills in addition to automatic scoring, level setting, interactive question forms, post‐operation feedback, Target in Tooth (a manual dexterity skills in which the goal is to remove a target volume, whilst removing as little as possible from the surrounding leeway (safety margin)), assessment of adjacent tooth and a self‐assessment tool to quantify the learning outcome of learners.^13^, ^14^

The literature reinforces consistent and timely feedback is essential for learners to make incremental improvements and transition to mastery.^9^

### 3.3 Pre‐brief

#### 3.3.1 Task design

This was facilitated by collaborative working between the e‐learning team, including a newly appointed Haptics teacher who was dentally qualified. The haptics teacher had analysed the existing simulation curriculum and identified how haptic could be integrated transitioning preclinical learners psychomotor skills training in both traditional simulation and haptic virtual reality environment.

Existing Simodont Cases were utilised which included simple manual dexterity tasks, transitioning to various cariology cases as well as various indirect vision exercises designed to induct students into the use of the mirror and various burs. In conjunction with the Electronic Resources Officer (Haptics), module leads and associated academic faculty, new haptics cases were created to mirror the existing curriculum.

The cases chosen were used by the novice learners in years 2 and year 3 BDS students. Year 2 pioneered the technology and each group was exposed to over 20 h of haptic teaching.

All students were encouraged to submit their first attempt at each new case, which provided us with a baseline reading of students’ ability, in order to build a data set. These data are intended to support the creation of an evidence‐based approach. Additionally, learners were sent surveys to capture information on self‐perception of confidence and the impact on learning experience. It is envisaged that such data could potentially influence key stakeholders in UK dental education regulation.

### 3.4 Briefing

#### 3.4.1 Faculty involvement, guided by learning outcomes and students’ experience

The e‐learning team and key discipline faculty such as the restorative module lead used a range of media including face‐to‐face didactic presentations, online recordings, instructional videos, online reading material for faculty and learners. The faculty were inducted through the training first followed by the learners.

Ideal preparation videos were recorded by the discipline lead, and then edited to be inclusive for different styles of learners, such as the addition of captions and subtitles, by the Electronic Resources team. All new cases included screenshots of key stages in the case descriptions shown on the haptic dental trainers, as well as appropriate photos and X‐rays of teeth used in the case.

Ideal preparation videos were uploaded to the educational e‐learning platform, QMPlus, the university Virtual Learning Environment.

Induction into the new haptics laboratory was provided to all faculty in a number of dedicated training sessions. These sessions gave faculty a brief overview of the haptic machine and its capabilities, an investigation of the models and cases available, before faculty undertook several pre‐prepared cases which highlighted the haptic feedback capabilities (enamel, dentine, pulp and caries all providing different haptic feedback.

Faculty teachers in specialist subjects such as endodontics, cariology and fixed prosthetics were encouraged to try existing cases in their speciality, in order to understand the potential of haptics and how it could be used in the future to create specific cases, which better match the curriculum. Support was offered to teaching staff in the creation of new cases and models and the electronic resources officer (haptics) continues to develop these new cases in conjunction with subject specialists.

There has been eagerness to enhance the learning experience and teachers have worked collaboratively sharing experiences and supporting each other. This has led to the creation of a haptics teaching group which has assisted in evaluation and reflective practice which has helped in the continuous iterative process of learning.

#### 3.4.2 Conduct a pre‐briefing prior to each simulation

A step‐by‐step procedural checklist was provided and an invitation to submit questions in advance from the students was enabled so that their cognitive load was reduced during the simulation‐based learning experience.

Each haptic session saw students cleaning each haptic machine thoroughly, before configuring each dental trainer's height and position in order to conform to student posture guidelines.

Students were asked not to work on the haptic dental trainers for more than 45 min without taking a break. Each case was described in detail by the subject specialist, and the ideal preparation video is made available for students to watch both in the haptic clinic and online via the virtual learning environment. A member of the electronic resources team and/or a member of the teaching faculty were present at each simulation session in order to provide technical support.

### 3.5 Simulation and feedback

#### 3.5.1 Provide immediate feedback and debriefing

Feedback has been set up to be delivered in many forms and can be verbal, written, haptic or visual cues. Reflection has been initiated via a workbook. A debrief session addresses the learning outcomes and other concerns that the learners encountered or that the facilitators witnessed during the simulation‐based exercise. Each case was designed so that feedback could be given to students during and after the completion of the task.

Many of the cases include on screen metrics and assessment tools which provide the students with real time feedback. For cases such as manual dexterity, instant feedback is given to students on their progress, providing them with information on carious material removed and the iatrogenic removal of adjacent healthy tooth tissue (called leeway margin in the Simodont haptics software). Haptic cases can also be configured to provide students with the opportunity for self‐reflection. The teacher station within the haptic clinic is able to view up to eight haptic dental trainers at once, and project this onto the classroom screen. This means that tutors and students are able to view progress on cases and easily identify students who are struggling with the task, or who excel.

A post‐session debrief addresses the learning outcomes and other concerns that the learners encountered or that the facilitators witnessed during the simulation‐based exercise.

### 3.6 Reflection and debrief

#### 3.6.1 Evaluation

Reflection has been continuous both in learning and at the completion of simulation‐based learning activities. All faculty involved in the development of the Haptics curriculum are constantly evaluating the components including time allocation, simulation modality choice and facilities. This has been done by regular monthly working group meetings which have facilitated the review process.

## 4 DISCUSSION

The development of haptic trainers for use in undergraduate and postgraduate clinical training offers potential solutions for challenges posed by limitations on patient treatment opportunities. The Covid‐19 pandemic posed significant challenges for dental schools globally.^15^ On 20 March 2020, the National Health Service (NHS) in England asked dental practices to avoid all aerosol generating procedures. Five days later, on 25 March 2020 NHS England said ‘All routine, non‐urgent dental care including orthodontics should be stopped and deferred until advised otherwise’.^16^ The recovery to pre‐pandemic patient service care levels has been difficult to achieve. Provision of patient clinical experience for undergraduate and postgraduate training was significantly interrupted putting huge pressure on students and faculty to prepare appropriately trained dental graduates. One of the potential solutions was believed by the educators to be advanced simulation but lack of funding made this an infeasible solution. Additionally, the UK dental regulator, the General Dental Council, in its education role is familiar with simulation‐based learning systems such as Phantom Head simulation which provides a safe and practical approach for pre‐clinical students to practise and gain the basic operative and core clinical concepts prior to the transition to delivering patient care. Advanced simulation such as Haptics is yet to gain pedagogical impact.

The Haptic Dental Trainers are expensive and typically educational providers struggle to justify the cost. The team at this teaching institution were fortunate in being able to obtain significant funding from the regional charity. The drive for investment in haptic training is not just the planning for the next pandemic but rather that there could be many other advantages. Provision of rare clinical scenarios, for example, the management of dental trauma, could be provided by simulation for repeated practice. Skills acceleration could be measured rapidly and objectively and provided to the learner in real time. The Simodont® haptic virtual simulators provide highly realistic feedback through the dental drill and instruments compared to the traditional approach with plastic teeth in ‘phantom heads’. There could be the opportunity to upload patient specific dental information and images to the haptic trainer so that a student could practise repeatedly a particular procedure planned for their patient.^17^

Haptic training in dentistry is increasingly used around the world,^18^ but yet there is a paucity of evidence on its pedagogical effectiveness. One recent review found that of the 36 included studies, the majority were cross‐sectional in design with short‐term evaluation data.^19^There is the view that haptic simulators are useful in early dental training, can accelerate skill learning and confidence, and complement the existing phantom head simulators. However, research in areas beyond these issues is scarce. Considering the financial investment required alone, there is a need to establish longer term benefits. It would be good to know for example, if simulation can replace time spent by learners in traditional phantom head training or patient clinics. Is there any measurable patient safety advantage? Answers to these types of questions are also important to provide evidence to funders and professional regulators in the UK and others globally.

In other fields in healthcare education, the use of Virtual, Augmented, and Alternate Reality have been found to have a variety of educational applications, improve surgical performance and impact on patient safety.^20^, ^21^, ^22^

The strategy adopted therefore in introducing haptic training to the curriculum at this teaching institution has been to ensure that consideration be given to doing this in a structured framework to comply with internationally recognised standards of best practice. It was important for us to ensure that development of the curriculum was aligned to an evidence‐based, best‐practice framework. The early adoption of this innovative simulation tool from both faculty and students has assisted in the accelerated integration into the curriculum. Faculty have been proactive in creating cases and in adapting the simulation curriculum to adopt Haptics. The positioning of the role of clinically trained haptics manager has helped in trouble shooting and also supporting faculty when challenges have arisen. Each member of faculty has been provided with the opportunity for one to one training and there has been complete uptake. This has been facilitated by flexible one to one tailored drop in sessions. These sessions have been labour intensive, however has been enabled by an extended timeframe of 6 months over which this has occurred. Haptics is a novel tool that has been perceived as useful in the early stages of integration. It is to be expected that as integration into the entire curriculum occurs, there are likely to be challenges which will need the school to adapt.

Early evaluation by students has suggested enhanced satisfaction and improved confidence following initial pre‐clinical haptics training. The restrictions of time in simulators has not been critiqued, however these are immediate findings.

## 5 CONCLUSION

Virtual reality haptics trainers have created unique opportunities and challenges for dental schools. Dental educators have sought to utilise this technology in a structured framework to enhance training**.**

## CONFLICT OF INTEREST STATEMENT

There are no conflicts of interest.

## Data Availability

The data that support the findings of this study are openly available in [repository name] at [DOI].
